# A review of stress-induced hyperglycaemia in the context of acute ischaemic stroke: Definition, underlying mechanisms, and the status of insulin therapy

**DOI:** 10.3389/fneur.2023.1149671

**Published:** 2023-03-21

**Authors:** Mengyue Yao, Yulei Hao, Tian Wang, Meizhen Xie, Hui Li, Jiachun Feng, Liangshu Feng, Di Ma

**Affiliations:** ^1^Department of Neurology and Neuroscience Centre, The First Hospital of Jilin University, Changchun, Jilin, China; ^2^Stroke Centre, Department of Neurology, The First Hospital of Jilin University, Changchun, Jilin, China

**Keywords:** stress-induced hyperglycaemia, acute ischaemic stroke, admission blood glucose, intensive glucose control, insulin

## Abstract

The transient elevation of blood glucose produced following acute ischaemic stroke (AIS) has been described as stress-induced hyperglycaemia (SIH). SIH is common even in patients with AIS who have no previous diagnosis of diabetes mellitus. Elevated blood glucose levels during admission and hospitalization are strongly associated with enlarged infarct size and adverse prognosis in AIS patients. However, insulin-intensive glucose control therapy defined by admission blood glucose for SIH has not achieved the desired results, and new treatment ideas are urgently required. First, we explore the various definitions of SIH in the context of AIS and their predictive value in adverse outcomes. Then, we briefly discuss the mechanisms by which SIH arises, describing the dual effects of elevated glucose levels on the central nervous system. Finally, although preclinical studies support lowering blood glucose levels using insulin, the clinical outcomes of intensive glucose control are not promising. We discuss the reasons for this phenomenon.

## 1. Introduction

Acute ischaemic stroke (AIS) is an acute brain injury that often occurs when an artery is suddenly blocked. It is one of the most common causes of severe disability and mortality worldwide (1). One description of stress-induced hyperglycaemia (SIH) is increased blood glucose due to a sudden clinical event that returns to baseline following the acute phase (2). SIH is commonly seen in AIS patients, even those with no history of diabetes mellitus (DM) diagnosis (3). Hyperglycaemia has been demonstrated to be independently linked to an adverse prognosis in AIS patients (4). Patients with previously undiagnosed DM are more likely to have increased short- and long-term mortality due to elevated blood glucose on admission to the hospital than patients with previously identified DM (3, 5). Hyperglycaemia has a dual effect on the central nervous system, with blood glucose above a specific range accelerating thrombosis, increasing stress and inflammatory responses, exacerbating reperfusion injury, and leading to lactate accumulation and mitochondrial dysfunction. This ultimately contributes to the transformation of the ischaemic penumbra into an infarcted region (6, 7). Therefore, it appears to be essential to maintain blood glucose levels in AIS. Although there are many published studies on SIH, there is no agreed-upon definition of SIH with AIS, such as admission blood glucose (ABG), stress hyperglycaemia ratio (SHR), or glucose variability (GV). The current large prospective controlled studies of SIH in patients with AIS treated with intravenous insulin have been designed using ABG as a definition and have not yielded desirable results (8–13).

Thus, in this review, we will discuss which optimal glycaemic definition of SIH is most associated with poor prognosis following AIS and whether the best definition cut-off value makes a difference in the presence and absence of recognized DM. Finally, we discuss whether insulin-intensive glucose control therapy can aid AIS patients with an improved prognosis.

## 2. Definition of stress-induced hyperglycaemia in acute ischaemic stroke

In the context of AIS, SIH is measured in different ways. However, it is still being determined which are the optimal glucose metrics for measuring SIH, as well as the definition and cut-off value of SIH that lead to adverse outcomes in AIS patients in the presence and absence of recognized DM (Table 1).

**Table 1:** The definition and a cut-off value of SIH related to adverse prognosis in ischaemic stroke patients with or without a previous DM diagnosis.

**Definition**	**Cut-off value (patients)**	**Primary relevant endpoint (in non-DM)**	**Primary relevant endpoint (in DM)**	**References**
ABG	110–126 mg/dl	In-hospital mortality; 30-day mortality	Not relevant	(3)
	130 mg/dl	Greater stroke severity; functional impairment; 90-day mortality	Not as strong as non-DM	(5)
	131.4 mg/dl	12-month poor functional outcomes	Same as non-DM	(14)
	140 mg/dl	Post-stroke infection; 3-month mortality; 3-month poor functional outcomes	-	(15)
	140 mg/dl (IVT)	Low complete recanalization rate; SICH; 3-month poor functional outcomes	Same as non-DM	(16)
	140.4 mg/dl (IVT)	3-month poor functional outcomes	Not relevant	(17)
	200 mg/dl (IVT)	7-day mortality; 3-month mortality; END		
	140 mg/dl (MT)	3-month poor functional outcomes; 3-month mortality; SICH	Same as non-DM	(18)
	140.4 mg/dl (IAT)	Poor functional outcomes at discharge	Same as non-DM	(19)
	140.4 mg/dl (IVT)	Not relevant with 3-month poor functional outcomes and SICH	Same as non-DM	(20)
	113.4 mg/dl	30-day case-fatality	-	(21)
	185.4 mg/dl	-	30-day case-fatality	
	113.5 mg/dl	In-hospital mortality	-	(22)
	210.5 mg/dl	-	In-hospital mortality	
Random blood glucose < 48 h	155 mg/dl	Admission stroke severity; 3-month poor functional outcomes; 3-month mortality	Same as non-DM	(23)
GAP	45 mg/dl	-	Stroke severity; poor neurological status	(24)
SHR	SHR1 ≥ 27.59			
	SHR2	1-year stroke recurrence; 1-year all-cause death	-	(25)
	SHR2	1-year poor functional outcomes; 1-year all-cause death	Same as non-DM	(26)
	SHR2	Haemorrhagic transformation	Same as non-DM	(27)
	SHR2	-	In-hospital mortality	(28)
	SHR2 (IVT)	3-month poor outcomes; 3-month mortality; SICH	Same as non-DM	(29)
	SHR2, SHR3 (IVT)	3-month poor functional outcomes	Same as non-DM	(30)
	SHR2 (IVT)	END and poor functional outcomes at discharge	Only END	(31)
	SHR2 (IVT)	3-month poor outcomes; 3-month mortality; SICH	Not relevant	(32)
	SHR2 ≥ 0.97 (IVT)	3-month poor functional outcomes	Same as non-DM	(33)
	SHR2 (MT)	3-month poor outcomes; 3-month mortality; SICH	Same as non-DM	(34)
	SHR3 ≥ 0.96 (MT)	3-month poor functional outcomes	Same as non-DM	(35)
GV	MAGE	-	END	(36)
	SD, CV	28-day, 90-day mortality	Same as non-DM	(37)
	SD (IVT and MT)	3-month poor functional outcomes	Same as non-DM	(38)
	MAG	Not relevant	3-month PSCI	(39)
	TR (MT)	3-month poor functional outcomes; SICH	Same as non-DM	(40)
	Max BG– min BG during hospitalization	-	3-month poor functional outcomes	(41)
	J-index	-	3-month increased cardiovascular events	(42)

–, Not mentioned.

### 2.1. ABG/FBG

Several earlier clinical studies defined hyperglycaemia in terms of the blood glucose value, or ABG, within 24 h of admission to the hospital for AIS patients. The cut-off values for ABG to define hyperglycaemia in AIS patients vary between studies.

Whether elevated ABG has different effects on the prognosis of AIS patients with and without a DM diagnosis has been widely discussed. In a meta-analysis of 32 cohort studies, Capes and colleagues (3) observed that non-DM patients with ABG above 126 mg/dl had a threefold increased admission and 30-day mortality compared to patients with normal ABG. In addition, the researchers found that ABG was unrelated to an increase in short-term mortality in DM patients. A prospective study (5) including 447 AIS patients suggests that, in non-DM patients, 90-day mortality was 3.4 times greater in patients with ABG above 130 mg/ dl than in those with normoglycaemia. In comparison, for DM patients, the hazard ratio was 1.6. Another observational study (15) of 2,550 AIS patients found that ABG ≥140 mg/dl was independently correlated with post-stroke infection in non-DM patients. In contrast, the same result was not found in DM patients. However, a series of studies suggested that ABG ≥140 mg/dl is strongly correlated with the risk of symptomatic intracranial hemorrhage (SICH) after intravenous thrombolysis (IVT), mechanical thrombectomy (MT) or intra-arterial treatment (IAT) and a poor 3-month clinical prognosis in AIS patients. These studies did not specifically distinguish between non-DM and DM patient populations (16, 18–20). A later study (17) carefully differentiated between these two groups and showed that ABG ≥140 mg/dl was related to an adverse prognosis after IVT in non-DM AIS patients. In conclusion, absolute increases in hyperglycaemia were more strongly related to short- or long-term mortality, post-stroke infection, and adverse prognosis in AIS patients with non-DM than in patients with previously diagnosed DM.

Furthermore, Snarska et al. (22) discovered that the cut-off values for ABG, which predicted the risk of in-hospital death, were distinct in AIS patients without and with DM (113.5 mg/dl and 210.5 mg/dl). This difference is consistent with that reported by Farrokhnia et al. (21) and has been found similarly in patients with myocardial infarction (43). The reason for this discrepancy may be that the baseline glucose metabolism levels are usually significantly higher in DM patients than in non-DM patients. The ABG values reflect both acute stress and chronic glucose metabolism levels.

In addition, several studies (44) have focused on fasting blood glucose (FBG), an alternative metric for ABG. Elevated FBG on admission following AIS was reported to be appreciably correlated with poor function only in pre-DM patients, with no correlation in DM patients. It is inconclusive to define SIH in terms of ABG or FBG without considering the prior glucose metabolic status. As a result, new indexes such as glycaemic gap (GAP) and SHR were introduced, eliminating the interference of chronic glycaemic levels.

### 2.2. GAP/SHR

GAP is calculated as the difference between ABG and long-term mean glucose values determined from HbA1c. In a study aimed at comparing the ability of SHR, GAP, and ABG to assess poor prognosis in AIS patients, Yang et al. (24) demonstrated that a GAP of 45 mg/dl showed a superior ability to differentiate between AIS patients' severity and prognosis compared to ABG, as did SHR.

SHR is measured as ABG or FBG divisible by long-term mean glucose values determined from HbA1c and is also measured as FBG divisible directly by HbA1c in some articles. An analysis (26) of 8,622 AIS patients reported an association between high SHR and severe neurological deficits and all-cause mortality at 1 year in patients with and without DM. Interestingly, the mortality outcome was more significant in patients without DM, as found in a nationwide prospective registry study among AIS patients with non-DM in China (25). A recent retrospective study (28) assessed the impact of SHR, FBG, and HbA1c on in-hospital mortality in AIS patients with DM. SHR appeared to have a better predictive value than other absolute measures. Ngiam and colleagues (33) discovered that high SHR, particularly at SHR ≥ 0.97, was strongly related to 3-mouth adverse outcomes in AIS patients after IVT with or without DM. Other studies have also found this correlation (29, 31). The same has been confirmed in AIS patients after MT (34, 35). In addition, some studies have shown that in the above calculation, SIH obtained by direct division of FBG by HbA1c correlates more with poor functional outcomes after IVT (30, 45).

### 2.3. GV

ABG and SHR cannot truly reflect the fluctuations in blood glucose under the influence of certain diseases due to the limitations of their numerical sources (obtained from HbA1c). Hyperglycaemic fluctuations caused by acute disease tend to exacerbate oxidative stress and damage endothelial cells, which results in a poor prognosis (46, 47). GV is the extent to which blood glucose levels fluctuate through time (48). An elevated GV is regarded as a sign of hypoglycaemia (49). There are two common types of GV, long-term with continuous blood glucose monitoring in years and short-term with days and months, and the most studied concerning stroke prognosis is short-term GV (50). Short-term GV is calculated in several ways, such as the mean amplitude of glycaemic excursions (MAGE), standard deviation, and coefficient of variation (50). In a 7.5-year follow-up of 28,354 patients with type 2 DM, the study observed that long-term high GV significantly enhanced stroke risk in DM patients (51). In a series of explorations of short-term GV, Hui et al. (36) found that altered GV in the first 3 days of hospitalization in AIS with DM patients was strongly related to early neurological deterioration (END) and that GV may be a more appropriate indicator than HbA1c. Yoon and colleagues (42) demonstrated that initial GV significantly enhanced the cardiovascular mortality risk at 3 months. In addition, elevated acute GV was related to poor functional outcomes (38, 41), impaired cognitive function (39), and a higher risk of haemorrhagic transformation (40), although GV was defined differently in these studies. GV has not been specifically studied in AIS populations with non-DM, possibly because diabetic populations are more likely to have measurable blood glucose changes.

In summary, we explored glycaemic indicators that could describe the full range of SIH following AIS. SHR has a similar predictor value for short- and long-term adverse outcomes in patients with and without DM and is independent of background glucose. Thus, SHR is expected to be a biomarker for SIH. Nevertheless, the current relevant studies do not provide a good head-to-head comparison of the different glucose markers. In the future, more prospective trials are needed to compare the clinical applicability of glycaemic indicators such as ABG, SHR, and GV or to attempt to apply them in combination.

## 3. Underlying mechanisms of SIH after AIS

**Figure 1:**
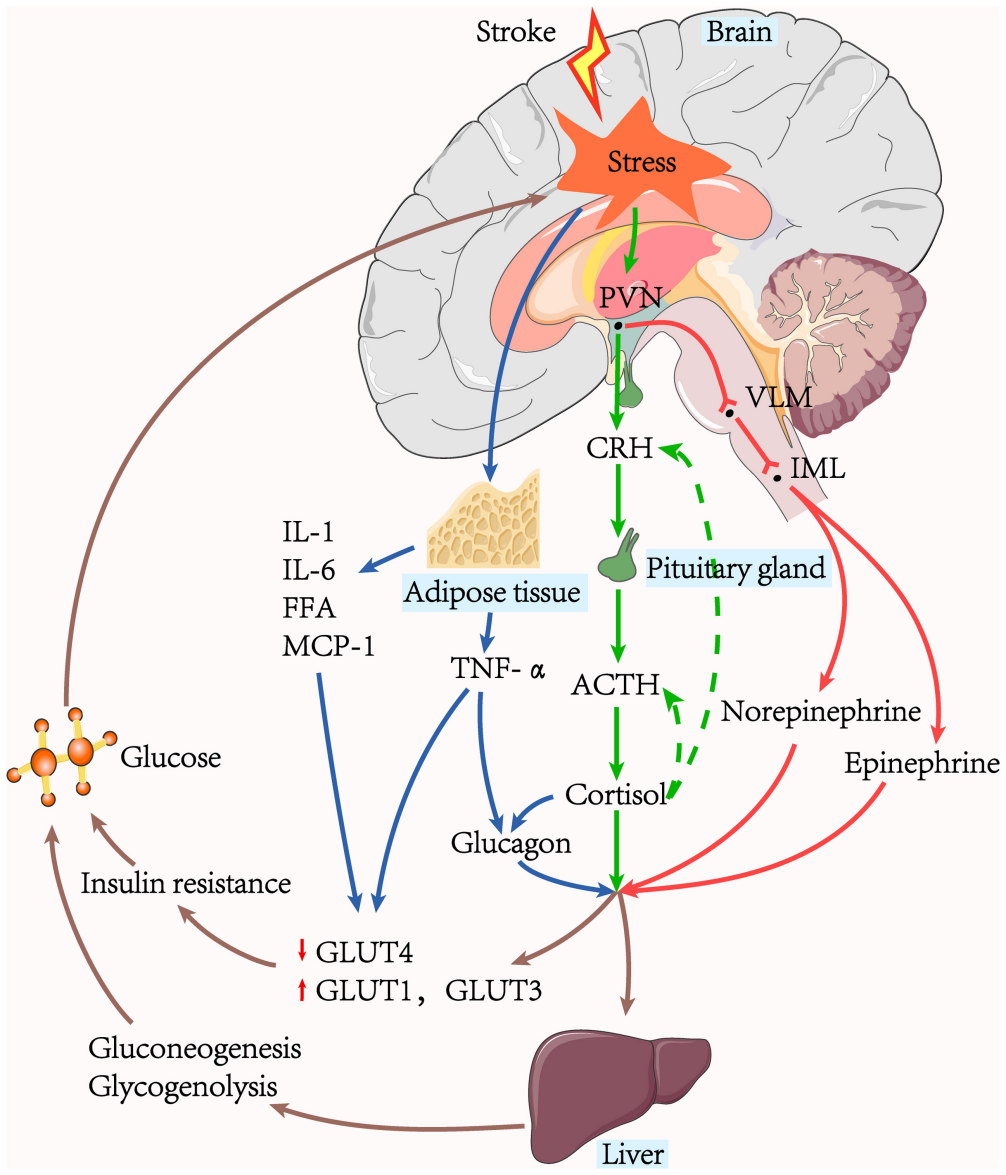
The pathway of stress-induced hyperglycaemia generation. Hepatic gluconeogenesis, glycogenolysis, and insulin resistance are the leading causes of hyperglycaemia. The sympathoadrenal system and the HPA axis are active in acute ischaemic stroke. The stressors stimulate the adrenal medulla to release catecholamine *via* the PVN-VLM-IML pathway, while the HPA axis stimulates the adrenal cortex to produce cortisol. Glucagon can be stimulated by cortisol, and TNF-α secreted from surrounding tissues. Glycaemic hormones such as catecholamine, cortisol, and glucagon, act on the liver to promote hepatic gluconeogenesis and glycogenolysis. Hyperglycaemia further exacerbates the stress response and contributes to the increased release of pro-inflammatory factors, creating a vicious cycle.

The combined synergistic effects of SIH in patients with AIS can be induced by the glucose regulatory center, the hypothalamic–pituitary–adrenal (HPA) axis, the sympathetic adrenomedullary system, and humoral factors (Figure 1).

### 3.1. Glucose regulatory center

The insular cortex controls the output of the sympathetic and parasympathetic nervous systems, and several studies have shown it to be associated with elevated blood glucose following acute ischaemia (52–54). Many brain regions, including the hypothalamus and brainstem, have also been demonstrated to alter blood sugar levels and stress responses. The catecholamine neuronal system in the brainstem, including the locus coeruleus, nucleus tracts solitaries, and ventrolateral medulla (VLM), significantly contribute to stress responses. Catecholamine neurons in the VLM are the control centers of SIH and receive inputs directly from multiple stress-responsive brain regions, including the hyperglycaemic excitability of the hypothalamic paraventricular nucleus (PVN)-VLM pathway (55). In addition, the preganglionic neurons of the sympathetic medullary system are located in the intermediolateral nucleus (IML) (56, 57).

### 3.2. HPA axis and the sympathetic adrenomedullary system

Gluconeogenesis, glycogenolysis, and excessive insulin resistance contribute significantly to the production and maintenance of hyperglycaemia during AIS (2, 58). Stressor stimulation causes excitation of the HPA axis and increases circulating cortisol. Cortisol has several metabolic effects to achieve elevated blood glucose levels, including activating vital hepatic gluconeogenesis enzymes and reducing glucose uptake in peripheral tissues (2). Stressor stimuli also converge on the brainstem catecholaminergic neurons and spinal cord efferent neurons in the medial column of preganglionic sympathetic neurons, activating the sympathetic adrenomedullary system and increasing blood levels of norepinephrine and epinephrine. Both epinephrine and norepinephrine stimulate the expression of essential genes that regulate glycogenolysis and gluconeogenesis (55). Norepinephrine additionally has the impact of increasing glycerol supply to the liver *via* lipolysis.

### 3.3. Humoral factors

#### 3.3.1. Hormones

Excess glucagon is the main mediator of glucose metabolism and can be stimulated by cortisol (59). Studies have shown that glucagon, catecholamine, and cortisol act synergistically in changes in glucose metabolism to rapidly raise fasting plasma glucose. Insulin is a naturally significant hormone that lowers blood glucose levels. Its action mechanism is mainly through the mobilization of cells in the liver, skeletal muscle, and other peripheral tissues to synthesize and store glycogen, fats, and proteins and reduce their catabolism (60). Under physiological conditions, the increase of the plasma insulin level stimulates the onset of glucose storage activity mediated by the insulin-sensitive glucose transporter (GLUT)-4, occurring primarily in muscle and adipose tissue, where GLUT-4 translocates from intracellular storage to the membrane, thus enhancing glucose uptake (61). However, following AIS, decreased insulin-mediated glucose uptake was paralleled with over-expression of the insulin-insensitive GLUT-1 and GLUT-3 in other tissues throughout the body, blocking GLUT-4-mediated glucose storage, further exacerbating the increase in peripheral blood glucose (2).

#### 3.3.2. Cytokines

Acute insulin resistance manifests as insulin-mediated glucose uptake reduction, mainly due to defective post-receptor insulin signaling and down-regulation of GLUT-4. Tumor necrosis factor-α (TNF-α), interleukin (IL)-1, and IL-6 can inhibit post-receptor insulin signaling, and TNF-α can also directly down-regulate the expression of the messenger RNA of GLUT-4 (62). An activated sympathetic nervous system induces adipocyte decomposition and increases free fatty acids (63). Excess circulating free fatty acids inhibit post-receptor insulin signaling and glycogen synthase to reduce glucose uptake (64, 65). In addition, fatty tissue secretes large amounts of pro-inflammatory cytokines, such as monocyte chemotactic protein-1 (MCP-1), an essential participant in insulin resistance (66, 67). TNF-α may also contribute to elevated blood glucose levels *via* the promotion of glucagon production (68).

## 4. SIH-induced protective effects after AIS

### 4.1. Phenomenon of protection

Glucose is a major energy supplier to brain tissue and provides a valuable metabolic substrate during the acute disruption of cerebral blood flow. In animal models of recirculation after ischaemia, ATP (indicating the recovery of energy metabolism) tended to increase in hyperglycaemia groups more than in hypo- and normoglycaemia groups (69). A study of rabbit models of focal cerebral ischaemia observed a smaller area of cortical brain infarction in the glucose-perfused group compared to the saline group (70). Moreover, an animal model of haemorrhagic shock demonstrated that rapidly induced hyperglycaemia resulted in a significant elevation in blood pressure, cardiac output, and viability (71). However, saline or mannitol at similar osmolar doses did not achieve the above effects. These studies suggest that elevated glucose rescues damaged brain tissue to some extent and improves survival.

### 4.2. Physical mechanisms of protection

Glucose diffuses along a concentration gradient from the bloodstream into the cells in the ischaemic area. Appropriate hyperglycaemia maximizes the guarantee of cellular metabolism. Uytenboogaart et al. (72) reported the concentration-effect phenomenon in lacunar stroke, where glucose values above 144 mg/dl were linked to a good functional prognosis. It is worth noting that hyperglycaemia worsens the clinical prognosis in non-lacunar stroke compared with lacunar stroke (73). This is possibly due to non-lacunar infarction of an area known as the ischaemic penumbra, the site of reduced blood flow around the ischaemic core. Hyperglycaemia causes cellular acidosis by increasing its intracellular lactate content, leading to a poor prognosis (74). In lacunar infarcts, this is absent, and elevated blood glucose can supply energy to the surrounding tissues, leading to a better prognosis. However, its beneficial effects are diminished when severe hyperglycaemia exceeds 216 mg/dl (72).

### 4.3. Biological mechanisms of protection

The physiological protective mechanisms of short-term increases in hyperglycaemia have been extensively explored in cardiac ischaemia (75, 76). Acute hyperglycaemia reduces post-ischaemic cell death by producing cell survival proteins, releasing cellular survival factors, and favoring angiogenesis. In a porcine coronary ischaemia-reperfusion model, Chu et al. (77) demonstrated that hyperglycaemia during acute ischaemia increased the production of cell survival proteins, such as phosphorylated endothelial nitric oxide synthase (eNOS) and heat shock protein 27 and thus reduced infarct size. Malfitano et al. (78) showed that hyperglycaemia decreased pro-inflammatory cytokines, increased cell survival factors (hypoxia-inducible factor-1α, vascular endothelial growth factor), and reduced apoptosis in the rat myocardial infarction model, thereby improving systolic myocardial function and reducing the size of the myocardial infarction. In addition, hyperglycaemia contributes to an increase in capillaries and a reduction in fibrosis. Subsequently, the protective effect of hyperglycaemia on ischaemic injury in myocardial infarction was also shown to increase antioxidant enzyme activity, improve glutathione redox balance, and reduce sympathetic activity after infarction (79).

## 5. SIH-induced damage after AIS

**Figure 2:**
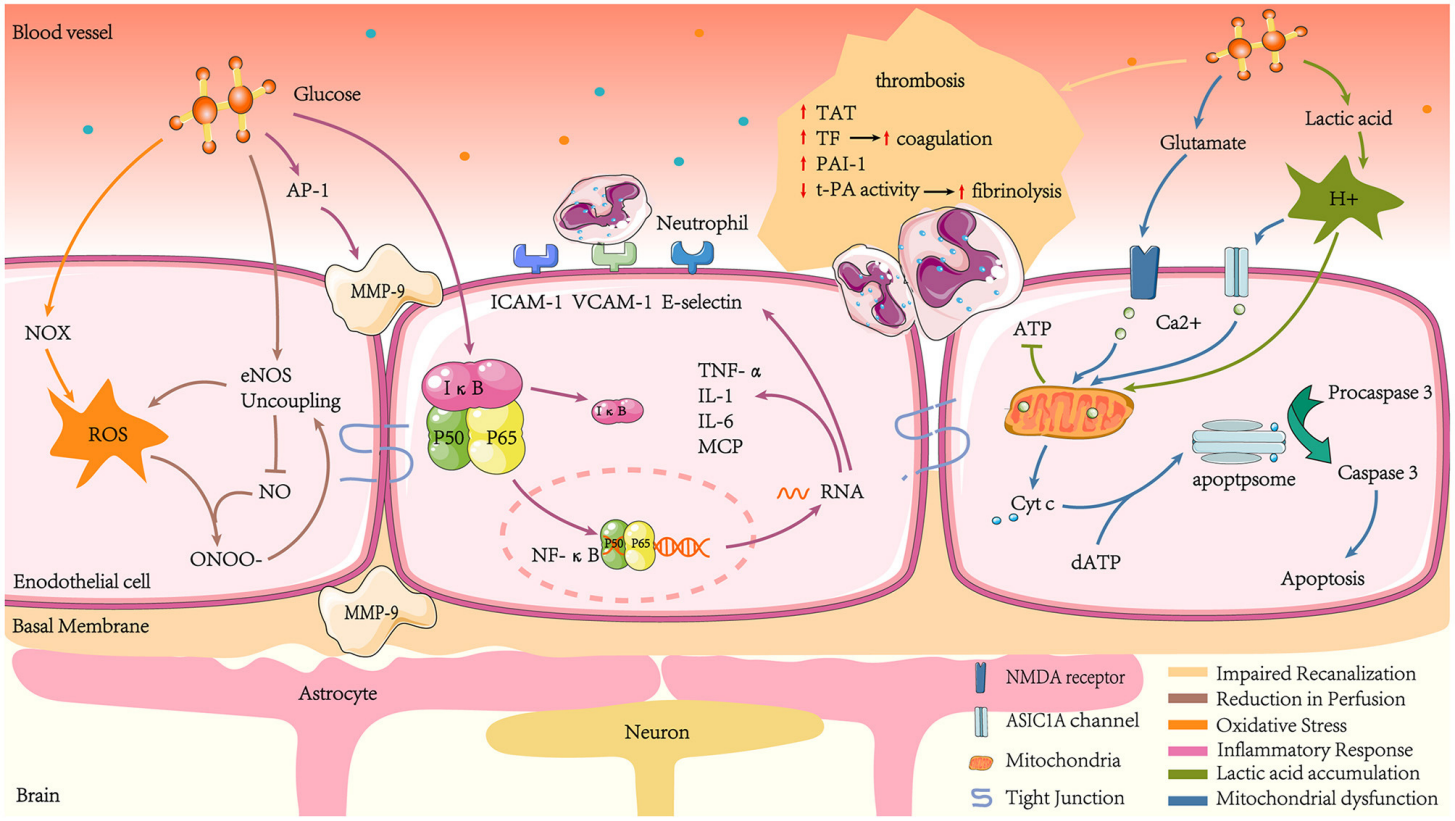
Mechanisms of hyperglycaemia-mediated damage in ischaemic stroke. Acute hyperglycaemia increases thrombosis by promoting coagulation and inhibiting hyperfibrinolysis, such as increased TAT, TF, and elevated PAI-1 activity, while t-PA activity decreases. Hyperglycaemia amplifies ROS production, which, together with NO, produces ONOO- and reduces NO bioavailability. At the same time, hyperglycaemia reduces NO production by promoting eNOS uncoupling, leading to endothelial cell diastolic dysfunction and reduced cerebral blood flow. Hyperglycaemia also increases NF-κB translocation to the nucleus by regulating IκB proteasomal degradation. NF-κB combines with specific κB sites on DNA sequence promoters to transcribe inflammatory factors such as TNF-α, IL-1, and IL-6. The NF-κB pathway also transcribes the chemokine MCP and adhesion factors such as ICAM-1, VCAM-1, and E-selectin, which induce leukocyte adhesion. Hyperglycaemia stimulates MMP-9 production, which can damage tight junctions, basement membrane proteins, and astrocyte peduncles, leading to BBB leakage. Hyperglycaemia exacerbates lactate accumulation in the ischaemic zone, leading to mitochondrial dysfunction and reduced ATP production, which fails to maintain intra- and extracellular osmotic and ionic gradients and mediates cytotoxic cell death. In addition, the overproduction of excitatory glutamate in response to hyperglycaemic stimulation activates NMDA receptors. At the same time, the lactate aggregation environment leads to increased H^+^ and promotes the opening of ASIC1 channels. Together, this results in increased intra-mitochondrial Ca^2+^. The imbalance of intra-mitochondrial Ca^2+^ exacerbates the leakage of Cyt c into the cytosol. Cyt c then interacts with dATP to activate downstream apoptogenic proteins, eventually triggering apoptosis.

The presence of acute hyperglycaemia leading to adverse prognosis has been repeatedly validated in patients with AIS, irrespective of a prior diagnosis of DM, suggesting a possible causal relationship. Various studies have extensively explored these potential mechanisms (7), which are discussed below (Figure 2).

### 5.1. Impaired recanalization

Impaired recanalization is associated with enhanced coagulation and reduced fibrinolytic activity (80). In human studies, hyperglycaemia elevates thrombin-antithrombin (TAT) complexes and tissue factor (TF) to produce procoagulant effects (81). Animal and cytological studies revealed that acute hyperglycaemia resulted in elevated plasminogen activator inhibitor type 1 (PAI-1) and decreased plasminogen activator (t-PA) activity levels, leading to a hypercoagulable state by affecting fibrinolytic homeostasis, which was confirmed in human studies (82, 83). Ribo et al. (84) found that during tissue-type t-PA-induced recanalization, acute hyperglycaemia was related to lower recanalization rates compared to chronic hyperglycaemia, suggesting that hyperglycaemia affects reperfusion in the ischaemic penumbra by impairing the fibrinolytic system.

### 5.2. Reperfusion reduction

A considerable number of studies have reported the phenomenon of cerebral blood flow reduction in hyperglycaemic animals (85–87). In an animal model experiment, acute hyperglycaemia-induced by intraperitoneal injection of glucose was linked to a 24% decrease in cerebral blood flow in rats. Following the intraperitoneal injection of mannitol, the plasma osmolarity increased as much as that observed following glucose injection. In contrast, cerebral blood flow decreased by only 10% (88). This suggests hyperglycaemia may deteriorate brain function by inhibiting compensatory blood circulation after ischaemic injury. The possible mechanism is acute hyperglycaemia promoting the formation of eNOS uncoupling phenomenon in the microvascular system surrounding the ischaemic region, leading to increased reactive oxygen species (ROS) production and decreased NO production (89). The reaction between ROS and NO can rapidly form ONOO–, which exacerbates eNOS uncoupling and produces a vicious cycle of oxidative stress. Reduced NO production and bioavailability leads to local vasodilation and impaired reperfusion (90).

### 5.3. Oxidative stress and inflammatory response

#### 5.3.1. Oxidative stress

Oxidative stress is a condition where the body is out of balance concerning oxidation and antioxidation, with a tendency to oxidize and produce large amounts of free radicals. ROS are mainly generated by the mitochondria and nicotinamide adenine dinucleotide phosphate oxidase (NOX) in biological systems (91). Notably, NOX is a major origin of ROS in neuronal cells after transient cerebral ischaemia-reperfusion (92). In a model in which endothelial cells were co-cultured with astrocytes to mimic the blood-brain barrier (BBB) environment, high glucose caused a marked increase in BBB permeability through enhanced oxidative stress. The mechanism was shown to be that high glucose enhanced NOX activity and expression levels (93). In addition, a neuronal cell culture experiment revealed that glucose provided the essential electron donor for ROS production in neurons after reperfusion (92). Excess reactive oxygen species lead to increased BBB permeability, brain oedema, and ultimately increased infarct size by peroxidising lipids, proteins, and nucleic acids (94–96). Furthermore, reactive oxygen species consume NO and generate a range of oxygen-free radicals and nitro compounds. The decrease in circulating NO levels leads to vascular endothelial relaxation dysfunction.

#### 5.3.2. Inflammatory response

Another effect following ischaemia-reperfusion that is significantly enhanced by hyperglycaemia is inflammation. A study in rat stroke models demonstrated that hyperglycaemia triggers a thrombo-inflammatory cascade response and amplifies and exacerbates middle cerebral artery occlusion (MCAO)-induced downstream microvascular thrombosis. The thrombo-inflammatory cascade begins immediately after MCAO in hyperglycaemic rats and continues throughout the reperfusion phase, accompanied by increased matrix metalloproteinase-9 (MMP-9), serotonin, and TAT complex (97). The increase in MMP-9 may be explained by hyperglycaemia promoting the expression of the pro-inflammatory transcription factor nuclear factor-activated protein-1 (AP-1) (98, 99). The increased MMP-9 is implicated in BBB destruction, resulting in plasma protein and inflammatory cell leakage, brain oedema formation, and a worse neurological prognosis (100, 101). Further investigations of hyperglycaemia-induced BBB leakage during ischaemia/reperfusion may be due to tight junction injury, basement membrane proteins, and astrocytic peduncles (97).

In addition, glucose causes an increase in transcription factors in the nucleus: nuclear factor-kappaB (NF-κB) (102). NF-κB is stably coupled to the inhibitory protein IκB in the cytoplasm. After phosphorylation and hydrolysis of IκB due to inflammatory stimuli, the heterodimers of p50 and p65 (the active form of NF-κB) undergo nuclear translocation. They are transcribed and translated into inflammatory proteins, such as TNF- α, IL-1, IL-6, and MCP-1, amplifying and maintaining the inflammatory response (103). Hyperglycaemia induces the promoter of the NF-κB subunit p65, causing an increase in p65 gene expression, which accelerates inflammatory factor production (104). Intercellular adhesion molecule-1 (ICAM-1), vascular cell adhesion molecule-1 (VCAM-1), and E-selectin are also induced by NF-κB (105, 106). These cytokines, chemokines, and adhesion molecules attract leukocytes to areas of ischaemia (107–109). Excessive leukocyte infiltration destroys the BBB, which causes irreversible infarction in the ischaemic penumbra.

### 5.4. Lactic acid accumulation

Lactic acid is a source of energy that is metabolized in the brain in the absence of energy (110, 111). Lactic acid accumulation is another risk factor caused by hyperglycaemia following a stroke. Compared with normoglycaemic animals, hyperglycaemia significantly increases lactate concentrations in ischaemic regions of a cat's brain, decreases high-energy phosphate and pH, and converts the ischaemic penumbra into infarct regions (112, 113). A human study found a similar phenomenon that hyperglycaemia in perfusion-diffusion mismatched patients led to enhanced brain lactate production, reducing rescue rates post-infarct penumbra (74). The cause of this phenomenon may be that the accumulation of lactic acidosis causes acidosis, damaging mitochondria and affecting ATP production, leading to depolarisation of cell membranes and the inability to maintain osmotic and ionic gradients inside and outside cells, ultimately leading to cytotoxic cell death (114).

### 5.5. Mitochondrial dysfunction

The brain's energy supply is disrupted during AIS, and hypoxia depolarisation at presynaptic terminals results in the liberation of excitatory neurotransmitters such as glutamate (115). A study suggested that hyperglycaemia enhances extracellular glutamate build-up during cortical ischaemia (116). N-methyl-D-aspartate (NMDA) receptors are typically ionotropic glutamate receptors and are essential mediators of neuronal death during cerebral ischaemia (117). Excitatory glutamate activates NMDA receptors, allowing Ca^2+^ to effectively enter the cell and mitochondria. Hyperglycaemia induces cell apoptosis following a stroke by increasing Ca^2+^ in the mitochondria, a vital link being the entry of cytochrome c (Cyt c) across the mitochondrial membrane into the cytosol (118). Upon entry into the cytosol, Cyt c interacts with procaspase-9, apoptotic protease-activating factor-1, and dATP to form an activation complex (119). This complex then drives caspase-3 activation, which ultimately induces apoptosis (120).

The glutamate non-dependent Ca^2+^ loading pathway also contributes to Ca^2+^ toxicity in ischaemia. Acid-sensitive ion channels (ASICs) are proton-gated ion channels activated by protons and widely exist in the central and surrounding system (121, 122). In the center, ASICs are primarily expressed in neurons and act as pH sensors to cause neuronal excitation. In physiological conditions, pH is relatively stable in brain tissue. However, massive anaerobic glycolysis of glucose during hypoxic-ischaemia leads to higher lactate accumulation in ischaemic tissue, and the pH drops rapidly (112). ASIC1A, a subtype of ASIC, can be activated by acidosis and conduct Ca^2+^. An animal experiment has confirmed that ASIC1A knock-out mice resist ischaemia and acid damage (123). Acidosis induces Ca^2+^ into cells via ASIC1, independent of the glutamate pattern, leading to increased intracellular Ca^2+^ concentration, which activation can trigger/regulate multiple cellular processes (124). In addition, during the early stage of ischaemia-reperfusion, hyperglycaemia hinders Ca^2+^ recovery during reperfusion, prolonging the existence of intracellular Ca^2+^ (125).

## 6. Laboratory recommendations for glucose control therapy

Several laboratory studies have extensively explored the effects on infarct volume of pre- and post-infarction insulin administration in global or focal ischaemic stroke. All the results have shown that insulin has a potent neuroprotective effect, reducing infarct size in crucial brain areas such as the hippocampus, striatum, and cerebral cortex (12, 126). In a transient forebrain ischaemia model, direct injection of insulin or insulin-like growth factor-1 (IGF-1) into the ventricles reduced ischaemic neuronal damage, suggesting that insulin can achieve neuroprotective results through direct interaction action with brain tissue, possibly in part through the IGF-1 receptor (127). In a transient focal ischaemic stroke model, the combination of insulin and glucose nullified most of the apparent protective effects (128), suggesting that insulin's significant neuroprotective effect could be achieved by lowering blood glucose, in agreement with the results of another experiment (129). Additional animal experiments have confirmed that possible mechanisms for the protective effects of insulin also include the regulation of neurotransmitters, the promotion of glycogen synthesis, and the prevention of neuronal necrosis and apoptosis (Table 2).

**Table 2:** Mechanism of the neuroprotective effect of insulin in acute ischaemic stroke animals during hyperglycaemia.

**References**	**Ischaemia model**	**Species**	**Results**
Zhu and Auer (127)	Transient forebrain 15 s	Rat	Insulin may be reducing the size of brain infarcts through the direct interaction of IGF-1 with brain tissue.
Shuaib et al. (130)	Transient forebrain 10 min	Gerbil	Insulin increases brain extracellular GABA levels.
Hamilton et al. (128)	Transient MCAO 2 h + hypotension	Rat	Insulin lowers blood sugar from 8–9mM to 3–4mM, reducing the area of infarction in the cerebral cortex and striatum.
Lanier et al. (131)	Transient forebrain 10 min + hypotension	Rat	Insulin may exert neuroprotective effects through the regulation of glycogen metabolism.
Sullivan et al. (132)	Cardiac arrest 10 min + reperfusion	Rat	Insulin resumes neuronal protein synthesis after cerebral ischaemia by inducing dephosphorylation of eukaryotic initiation factor 2α.
Guyot et al. (133)	Transient global 10 min	Rat	Insulin increases brain extracellular GABA levels resulting in neuron inhibition.
Hui et al. (134)	Transient global 15 min	Rat	Insulin exerts neuroprotective effects by activating PI3K/Akt negative regulation of the JNK signaling pathway.
Sanderson et al. (135)	Transient forebrain 10 min + hypotension	Rat	Insulin activates the PI3K-Akt survival pathway.
Fanne et al. (136)	Transient MCAO 2 h	Rat	Insulin exerts a neuroprotective effect by lowering glutamate levels.
fan et al. (137)	Embolic focal strokes (blood clot)	Rat	Insulin glucose control during t-PA treatment reduces plasma PAI-1 levels and activities.
Sanderson et al. (138)	Transient forebrain 8 min + hypotension	Rat	Insulin prevents apoptosis upon entry of Cyt c into the cytosol by promoting Cyt c Tyr97-phosphorylation.
Hung et al. (139)	Transient forebrain 1 h + MCAO permanently	Rat	Insulin arrests NO reaction with superoxide to form peroxynitrite.
Huang et al. (140)	Transient forebrain 1 h + MCAO permanently	Rat	Insulin increases cerebral Akt/eNOS phosphorylation and improves neurologic function.
Ahmadi-Eslamloo et al. (129)	Transient MCAO 1 h+ reperfusion	Rat	Insulin attenuates focal brain tissue damage after ischemia-reperfusion in diabetic rats through hypoglycemic effects.

Transient forebrain, bilateral carotid occlusion plus reperfusion; Transient global, four-vessel occlusion plus reperfusion.

## 7. Clinical evidence of intravenous insulin therapy

### 7.1. Clinical practice of intravenous insulin therapy

Intensive glucose control with intravenous insulin effectively reduces in-hospital mortality in critically ill non-stroke patients (141, 142). Thus, in recent years, some clinical trials have explored the optimal glycaemic targets for intensive insulin treatment in AIS patients with elevated ABG (Table 3).

**Table 3:** Randomized trials aimed at comparing the effectiveness of intensive glycaemic treatment with standard treatment in acute ischaemic stroke patients with enhanced admission blood glucose.

**Patients (*n*)**	Admission blood glucose (mg/dL)^*^	**Intervention (time of treatment)**	**Mean blood glucose (mg/dl)**	**The difference in mean blood glucose after treatment (mg/dl)**	**Primary and relevant endpoint**	**References**
AIS < 12 h (24)	264 ± 88	Intravenous insulin + meal-related subcutaneous insulin (72 h)	131 ± 20	Not reported	Feasibility of insulin infusion	(143)
AIS < 24 h (13)	190.8 ± 34.2	Intravenous insulin (48 h)	124.5 ± 34.2	Not reported	Support insulin treatment within 48 h, not outside 48 h	(144)
All strokes < 24 h (460)	140.4 [112.4–165.6]	Intravenous GKI (24 h)	Not reported	10	3-month mortality, avoidance of severe disability or severe functional impairment (*N*)	(8)
AIS < 12 h (31)	259.2 ± 79.2	Intravenous insulin + meal-related subcutaneous insulin (72 h)	133	57	Feasibility and tolerability of insulin infusion; 90-day Functional outcomes (*N*)	(9)
AIS < 24 h (25 moderate glycaemic control; 24 tight glycaemic control)	167.4 [127.8–221.4]	Intravenous insulin (5 days)	111	Not reported	Feasibility and safety of insulin infusion	(145)
	167.4 [126–228.6]	Intravenous insulin (5 days)	151			
AIS < 24 h (20)	136.8 ± 84.6	Intravenous insulin (5 days)	116.8 ± 39.42	27.36	Feasibility and safety of insulin infusion	(146)
AIS < 24 h (10 insulin and tube feeding; 13 insulin only)	163.8 ± 43.2	Intravenous insulin + continual tube feeding (5 days)	104.4 ± 5.4	Not reported	Safety of intravenous insulin + continuous tube feeding	(147)
	189 ± 59.4	Intravenous insulin (5 days)	136.8 ± 27	Not reported		
AIS < 24 h (13 and 10 strict glycaemic control)	9.6 (7.3–18.6)	Intravenous insulin (5 days)	The target range of glucose control is 79.2–109.8	Not reported	Safety of intravenous and subcutaneous insulin in intermittently fed AIS patients	(148)
	8.6 (7.3–13.2)	Subcutaneous insulin (5 days)				
AIS < 24 h (25)	149.6 ± 50.22	Intravenous GKI (24 h/48 h/72 h)	6 h: 97.2	Not reported	Infarct growth at 7 days (*N*); ↓Brain lactate levels	(10)
			12 h: 104.4			
AIS < 12 h (26)	149 ± 16	Intravenous insulin (24 h)	88 ± 9	Not reported	Improved 30-day neurologic status; 24-hour and 30-day functional outcomes, 30-day mortality (*N*)	(11)
AIS < 6 h (87)	120.6 [109.8–140.4]	Intravenous insulin (24 h)	126	Not reported	Improved glucose control; ↑Infarct growth at 7 days; 90-day functional outcomes, mortality, serious adverse events (*N*)	(12)
AIS < 12 h (581)	188 [153–250]	Intravenous insulin (72 h)	118	61	90-day functional outcomes (*N*)	(13)

* Figures denote mean ± SE, median [inter quartile range]; n, number of non-diabetic patients receiving glucose-lowering treatment as a percentage of total; N, no difference compared to conventional treatment group.

A total of 933 patients with acute stroke (ischaemic or haemorrhagic) were enrolled in the GIST-UK trial within 24 h (8), 80% of whom did not have a diagnosis of DM and had a median ABG of only 140.4 mg/dl. The experimental group maintained capillary glucose at 72–126 mg/dl by 24-h continuous intravenous infusion of glucose-potassium-insulin (GKI). Surprisingly, compared to the control group, the 90-day functional outcomes of the experimental group did not improve, and hypoglycaemic events occurred. By analyzing changes in blood glucose and blood pressure over 24 h, the benefits of lowering blood glucose may have been masked by lower blood pressure. The study was criticized for including a heterogeneous stroke type, with slow recruitment, late treatment initiation, and variability in glycaemic control of only 10 mg/dl (12, 13). Subsequent studies assessing the feasibility and safety of intensive glucose control with insulin after AIS have shown that such trials are feasible and necessary (9, 144–146).

In the SELESTIAL trial (10), 30% of AIS patients with a previous DM diagnosis were maintained on serum glucose between 72–126 mg/dl by GKI infusion. Brain lactate production level decreased at 6–12 h following insulin administration. However, insulin treatment was linked to a marked increase in cerebral infarct size in patients with complete intracranial vascular occlusion. In addition, 76% of patients in the experimental group developed asymptomatic hypoglycaemia. Similarly, a large study explored the effects of an intensive glycaemic regimen compared with usual care on glycaemic control and infarct size expansion. In the INSULINFARCT trial (12), 180 AIS patients were randomized within 6 h to receive 24 h of intravenous insulin therapy, with the vast majority of subjects having no history of DM. The study found that keeping blood glucose below 126 mg/dl did not prevent the transition from an ischaemic penumbra to an infarcted area.

Since all of the above studies failed to demonstrate a positive effect of intensive glucose therapy on stroke prognosis, Johnston and colleagues began to consider that variability in glycaemic control and duration of treatment may be the problem. In the SHINE trial (13), a large multicenter randomized controlled trial, 1,151 AIS patients underwent conventional (80–180 mg/dl) or strict glycaemic control (80–130 mg/dl) within 12 h. Of the enrolled patients, 68% had received reperfusion therapy, and 80% were diagnosed with DM. The change in glycaemic control compared to GIST-UK was significant (61 mg/dl). Intensive glucose control at up to 72 h did not significantly improve 90-day functional outcomes.

### 7.2. Discussion on the ineffectiveness of insulin therapy

In reviewing the above clinical trials of intensive insulin glucose-lowering, only two studies distinguished between the two populations of AIS patients, with or without previously recognized DM, and discussed treatment prognosis. One study in AIS patients with DM revealed that maintaining blood glucose below 130 mg/dl with intensive insulin treatment was feasible but did not improve the prognosis at three months (9). In another clinical study of mild hyperglycaemia in non-DM patients after AIS, it was found that maintaining blood glucose levels at 81–126 mg/dl was relatively safe in the experimental group and improved neurological status after 30 days. However, there was no difference in functional outcomes compared to controls (11). The SHINE trial (13) did not yield the desired results. Torbey et al. (149) considered whether the different definitions of SIH determined the outcome. They performed a further subgroup analysis of SHINE, carefully selecting six glycaemic parameters: ABG, absence versus presence of diagnosed and undiagnosed DM, HbA1c, GAP, SHR (ABG/ average HbA1c-based daily blood glucose), and GV (SD). The results identified that patients with undiagnosed DM had the lowest likelihood of a good prognosis compared to those with true non-DM. However, no apparent benefit of SHINE treatment was observed in the small group of undiagnosed DM patients. After adjusting for baseline stroke severity and thrombolytic therapy, the researchers still found no benefit of intensive insulin therapy in any of the above subgroups. This study was a secondary analysis of the SHINE trial, and the results had unavoidable limitations. Because ABG cut-off values associated with poor prognosis are not the same in AIS patients with and without previously recognized DM, we suggest that future clinical studies should consider the different glycaemic backgrounds of the two populations, whose criteria for initiating glucose lowering, time of initiation and duration of glucose-lowering, and even glucose lowering goals may not be the same. Each characteristic needs to be determined by relevant basic and clinical trials. Next, we discuss the results based on the available experiments.

#### 7.2.1. Glucose reduction criteria

There is considerable heterogeneity in ABG treatment criteria in each study reporting functional outcomes, for example, 140.4 mg/dl (8), 259.2 mg/dl (9), 149 mg/dl (11), 120.6 mg/dl (12), and 188 mg/dl (13). Subsequent studies should include patients with glucose values above the cut-off value related to a worse prognosis, which is considered to be approximately 155 mg/dl (23). For patients treated with IVT or MT, or IAT, it is recommended to use ~140 mg/dl (16, 18, 19). Also, for non-DM patients with post-AIS hyperglycaemia, we recommend a glucose reduction criteria of ~126 mg/dl (3).

#### 7.2.2. Start time and duration of glucose lowering

In studies exploring the effects of intensive glucose control, glucose lowering was initiated at 6 h (12), 12 h (9, 11, 13), and 24 h (8) after stroke. The duration of glucose lowering ranged from 24 to 72 h. Rosso and colleagues (12) found that in AIS patients who underwent intensive insulin treatment immediately within 6 h, baseline and day 7 MRI comparison revealed a more significant infarct growth in the experimental group. Therefore, intensive insulin treatment is not recommended in the hyperacute phase of cerebral infarction. The proper duration of glucose lowering after AIS is still being determined. Data obtained from continuous glucose monitoring indicate that continuous glycaemic control for 72 h after AIS is necessary (150–152).

#### 7.2.3. Glucose-lowering targets

Glucose-lowering targets are controversial. In a rodent model of focal ischaemia, insulin treatment after ischaemia resulted in minimal infarct size between 108–126 mg/dl glucose and increased infarct size between 36–54 mg/dl glucose (153). However, in two subsequent clinical trials measuring the controversial infarct size increase, serum glucose was kept below 126 mg/dl using insulin infusion. One of these trials did not find any marked variation in infarct size increase over 1 week between the intervention group and the control group (10). In the other trial, the intervention group's infarct size was more remarkable (12). Furthermore, in a study of patients with AIS, ABG values between 66.6–131.4 mg/dl were associated with 12-month positive functional outcomes (14). Gentile et al. (154) found a 4.6-fold lower in-hospital mortality rate in AIS patients who controlled their blood glucose below 130 mg/dl within 48 h of admission compared with those experiencing persistent hyperglycaemia. However, several clinical studies have shown similar mortality and neurological prognosis in intensive insulin therapy (118–133 mg/dl) and conventional insulin therapy groups (8, 9, 12, 13). There are no consistent recommendations for optimal glycaemic targets for SIH in treating AIS due to limited clinical trial results. Current American Heart Association/American Stroke Association (AHA/ASA) guidelines for AIS recommend treating hyperglycaemia to stabilize blood glucose levels at 140 to 180 mg/dl (155). However, most guidelines endorse that tight glycaemic control (blood glucose < 126 mg/dl) or hypoglycaemia is harmful and should be avoided (12, 156).

#### 7.2.4. Hypoglycaemia

A recent meta-analysis on selecting an optimal glucose-lowering target for critical patients showed that glucose control levels < 110 and 110–144 mg/ dl had a greater hypoglycaemia incidence vs. 144–180 and >180 mg/dl (157). Hypoglycaemia exacerbates brain damage after AIS by increasing oxidative stress and inflammatory response. Studies on rodent models have shown that repeated hypoglycaemia increases post-ischaemic brain injury in diabetic rats by increasing mitochondrial ROS production and decreasing mitochondrial complex I activity after ischaemia (158, 159). In addition, hypoglycaemia induces increased IL-6 production, platelet aggregation, vascular adhesion molecule production, and inhibition of fibrinolytic mechanisms (160–162). These changes eventually lead to vascular injury and new thrombosis, increasing the risk of local cerebral ischaemia. Therefore, repeated hypoglycaemia has been indicated as an essential reason for the unsuccessful intensive glucose-lowering therapy. Future trials should be aimed at controlling blood glucose to “broad” levels rather than intensively lowering glucose to avoid harm from irreversible hypoglycaemic events.

### 7.3. Other hypoglycaemic drugs

#### 7.3.1. GLP-1 receptor agonists

Glucose-like peptide-1 (GLP-1) receptor agonists include exenatide, liraglutide, albiglutide, semaglutide, and dulaglutide considered to be the first alternative to insulin. GLP-1 is a peptide hormone secreted by the intestine following food stimulation and is implicated in regulating blood glucose homeostasis in the body. Natural GLP-1 is degraded by the endoprotease dipeptidyl-peptidase-4 (DPP-4) *in vivo* with an ~2-min half-life. GLP-1 receptor agonists can perform the same biological actions as natural GLP-1 but also avoid degradation and loss of activity, thus prolonging the duration of action. GLP-1 maintains glucose endocrine homeostasis through several mechanisms, including but not limited to promoting insulin secretion from pancreatic β-cells, inhibiting glucagon production, improving β cell quality and function, and decreasing appetite and gastric emptying. GLP-1 receptor agonists rarely cause hypoglycaemia, and their main drawback is mild to moderate gastrointestinal adverse effects. Findings from cardiovascular prognostic studies and meta-analyses suggest that GLP-1 receptor agonists reduce stroke incidence and have neuroprotective effects in DM patients (163). Some studies highlight that the neuroprotective effects of these drugs in AIS patients may be through the improvement of ischaemia-induced inflammation, support of BBB integrity by triggering the PI3K/AKT and mitogen-activated protein kinase (MAPK) pathway, as well as reducing ROS levels in ischaemic neurons (164, 165). The effectiveness and safeness of GLP-1 receptor agonists for treating hyperglycaemia after AIS has been demonstrated with exenatide at 9 h after AIS and continuing for 6 days to lower blood glucose (166). A recent multicenter randomized trial was designed to compare the difference in risk of hypoglycaemia and improvement in neurological prognosis with exenatide vs. standard of care. Exenatide 5 μg was administered subcutaneously twice daily in the treatment group, but the trial results have not yet been published (167).

#### 7.3.2. DPP-4 inhibitors

Another recommended alternative therapy is DPP-4 inhibitors, which exert hypoglycaemic effects mainly by inhibiting the physiological degradation of GLP-1 in the blood, including alogliptin, linagliptin, saxagliptin, sitagliptin, and vildagliptin. Interestingly, DPP-4 inhibitors improve neurological prognosis after stroke in rodents, independent of GLP-1, possibly by increasing the bioavailability of other bioactive DPP-4 substrates, such as stromal cell-derived factor-1α (167). In addition, preclinical studies have found that these drugs can activate the Akt/mTOR pathway and anti-apoptotic and anti-inflammatory mechanisms to exert neuroprotective effects (168). However, two recent extensive randomized clinical studies have shown that DPP-4 inhibitors are ineffective in reducing ischaemic stroke risk following cardiovascular disease (169, 170). This is inconsistent with the neuroprotective conclusions drawn from preclinical studies and requires further experimental validation.

#### 7.3.3. SGLT2 inhibitors

In addition to the two drugs mentioned above, the new glucose-lowering drug type, sodium glucose-linked cotransporter 2 (SGLT2) inhibitors, are recommended. Besides reducing blood glucose, SGLT2 inhibitors have antioxidant, anti-inflammatory, insulin resistance, and atherosclerotic plaque formation-reducing properties (171). In addition, SGLT2 inhibitors reversed hyperglycaemia-induced neuronal damage after acute cerebral ischaemia by inhibiting SGLT in a mouse model of temporary bilateral carotid stenosis (172). However, the current debate on whether SGLT2 inhibitors prevent strokes after cardiovascular disease is divided. In the EMPA-REG OUTCOM trial (173), there was a slight increase in stroke risk with empagliflozin. Some have attributed this trend to an elevated haematocrit in the empagliflozin group, which corresponds to an increased stroke risk due to increased blood viscosity. Fortunately, no such trend was found in the CANVAS trial (174). However, a meta-analysis concluded that SGLT2 inhibitors increased non-fatal stroke risk by 30% (175).

#### 7.3.4. Metformin

Metformin, a biguanide derivative, is the principal drug indicated for type 2 DM (176). The hypoglycaemic pharmacological effects of metformin are mainly through reducing hepatic glycogenolysis and enhancing peripheral tissue glucose uptake and utilization (177). Studies have shown that metformin can exert post-stroke neuroprotective effects, possibly inhibiting ischaemia-induced neuronal death, oxidative stress, and inflammatory responses (178, 179). The opening of the mitochondrial permeability transition pore (MPTP) is a significant cause of neuronal mortality due to ischaemia (180). Metformin may prevent neuronal death by inhibiting mitochondrial respiratory chain complex I, preventing MPTP opening, and activating AMPK (181, 182). Furthermore, metformin was demonstrated to regulate AIS-induced oxidative stress damage and reduce markers of brain inflammation *via* the lncRNA-H19/miR-148a-3p/Rock2 axis (183, 184).

The drawbacks of metformin compared to intravenous insulin application in critical care patients are apparent. First, compared with the immediate hypoglycaemic effect of insulin, the blood dose concentration peaked approximately 3 h following oral metformin (185). It fails to adjust rapidly to the dramatic changes in blood glucose concentration in patients. An exploratory safety study of metformin in patients with the transient ischemic attack or minor ischaemic attack found that 20% of patients in the experimental group permanently discontinued the drug due to gastrointestinal side effects (186) and its metabolic characteristics of being excreted mainly through the kidneys (185), which both limit its application. Finally, intravenous insulin can be quickly adjusted to each patient's diet, facilitating the maintenance of blood glucose stability during hospitalization. Other oral hypoglycaemic agents are available for similar reasons, such as sulfonylureas, glinides, α-glucosidase inhibitors, thiazolidinediones, and DPP-4 inhibitors. Therefore, intravenous insulin application seems to be a more appropriate treatment option than oral agents, such as metformin, in the case of elevated blood glucose after AIS.

## 8. Conclusion and perspectives

In this review, we discuss the definition of SIH in the background of AIS, its formative mechanisms, and its dual impact on the central nervous system. Insulin is currently a widely accepted clinical treatment for post-AIS hyperglycaemia, but it does not achieve the desired effect in practice. In our analysis of intensive insulin treatment failure, the best definition of SIH should be independent of the existence of DM and strongly linked to short- and long-term efficacy. SHR, or the application of these definitions in combination, is a good option for future studies and needs further exploration. In addition, the treatment of SIH should consider the criteria for initiating glucose lowering after AIS, the timing of dosing and duration of treatment, the target range of glycaemic control, and treatment options other than insulin. GLP-1 receptor agonists are a potential new research target for treating SIH.

## Author contributions

MY and YH reviewed the literature and wrote the draft manuscript. TW helped assess the literature. MX, HL, and JF drew the pictures. LF designed and revised the work. DM supervised the work. All authors contributed to the article and approved the submitted version.
